# A scientometrics analysis of physical activity and transcranial stimulation research

**DOI:** 10.1097/MD.0000000000035834

**Published:** 2023-11-24

**Authors:** Angel Denche-Zamorano, Noelia Mayordomo-Pinilla, Sabina Barrios-Fernandez, Vicente Luis-del Campo, Santiago Gómez-Paniagua, Jorge Rojo-Ramos, Antonio Castillo-Paredes, Laura Muñoz-Bermejo

**Affiliations:** a Promoting a Healthy Society Research Group (PHeSO), Faculty of Sport Sciences, University of Extremadura, Caceres, Spain; b Promoting a Healthy Society Research Group (PHeSO), Faculty of Sport Sciences, University of Extremadura, Caceres, Spain; c Occupation, Participation, Sustainability and Quality of Life (Ability Research Group), Nursing and Occupational Therapy College, University of Extremadura, Cáceres, Spain; d Laboratorio de Aprendizaje y Control Motor, Facultad de Ciencias del Deporte, Universidad de Extremadura, Cáceres, Spain; e BioẼrgon Research Group, University of Extremadura, Cáceres, Spain; f Physical Activity for Education, Performance and Health, Faculty of Sport Sciences, University of Extremadura, Caceres, Spain; g Grupo AFySE, Investigación en Actividad Física y Salud Escolar, Escuela de Pedagogía en Educación Física, Facultad de Educación, Universidad de Las Américas, Santiago, Chile; h Social Impact and Innovation in Health (InHEALTH), University Centre of Mérida, University of Extremadura, Mérida, Spain.

**Keywords:** brain, cognition, health, neural entrainment, sports performance

## Abstract

**Background:**

The search for alternatives to improve physical performance is rising, and in recent years has been focused on the brain. No bibliometric study analyzing research on physical activity (PA) and transcranial stimulation has been found in the scientific literature. Aims: To provide an overview of the existing scientific research on PA and transcranial brain stimulation in healthy and sports participants, using a bibliometric analysis and graphic mapping of the references in the field. To do this, we analyze annual publication trends in this area, identifying the most productive and cited authors, journals and countries with the highest number of publications, and the most cited documents and keywords.

**Methods:**

Those publications related to this area, published in journals indexed in the web of science main collection were retrieved and analyzed using the traditional laws of bibliometrics.

**Results:**

A total of 305 documents were found. Annual publications followed an exponential growth trend (R^2^ = 94.2%); with A. J. Pearce (9 documents) is the most productive coauthor and M.C. Ridding, H. Theoret and M. Lassonde as the most prominent (with 5 most cited papers). The USA (67 papers) and the journal Frontiers in Human Neuroscience (12 papers) were the most productive country and journal respectively. The paper “Action anticipation and motor resonance in elite basketball players” was the most cited paper and “transcranial magnetic stimulation” was the most used keyword.

**Conclusion:**

There are extensive research networks throughout the world, with the USA leading the production. Publications on the issue are of high interest in the scientific community as an exponential increase in publications over the last few years was found. The contribution of these findings is to offer a complete picture of the relationship between PA and transcranial brain stimulation in healthy individuals and athletes. Therefore, this comprehensive analysis provides fruitful information for sports researchers and policymakers to make future correct decisions about how to better design and implement training interventions in these groups of individuals.

## 1. Introduction

Exercise and physical performance are influenced by physiological, cognitive, emotional and social factors.^[1,2]^ Thus, a variety of ergogenic aids has been used for maximizing outcomes.^[3]^ In recent years, efforts in the search for alternatives to improve physical performance have been focused on the brain and how its stimulation improves physical performance. Non-invasive brain stimulation (NIBS) has been studied to induce transitory and controlled changes in brain activity to study its effects on motor, cognitive or perceptual processes. Colzato et al (2017)^[4]^ concluded that noninvasive brain stimulation techniques are promising tools to improve mental but also physical performance in athletes although they are not a common practice in sports competitions. The 2 most commonly used techniques are transcranial magnetic stimulation (TMS) and transcranial electrical stimulation (tES).^[5,6]^ TMS and tES are considered effective in disorders such as depression,^[7]^ pain^[8,9]^ or Parkinson disease^[10]^ among others, Although their efficacy in concussion^[11]^ or stroke^[12]^ is not yet well established. However, TMS and tES should be considered as support in the rehabilitation protocols (e.g., reinforcing improvements in the patients´ recovery) rather than the main approach. Various studies have focused on transcranial stimulation effects in improving cognitive functions, motor performance and physical performance.^[13–15]^ Indeed, transcranial stimulation has generally been used to modulate performance in perceptual-cognitive processes, such as response inhibition^[16–18]^ memory^[19–21]^ or reducing mental fatigue.^[22]^

On the one hand, TMS is a well-established and validated technique for quantifying excitation and inhibition in the primary motor cortex, spinal nerve roots or peripheral motor (corticospinal) pathway. TMS employs time-varying magnetic fields that induce electrical currents in the conducting neural tissue, being considered a neurostimulation technique.^[13]^ Then, when it is applied to the motor cortex, the response is recorded and measured as a motor-evoked potential on the target muscle electromyogram.^[5,23,24]^ On the other hand, tES applies current to electrodes on the scalp^[8]^ and it is considered a neuromodulatory technique.^[25]^ Transcranial alternating current stimulation, transcranial direct current stimulation (tDCS), and transcranial random noise stimulation are the most common tES forms.^[26,27]^ Most studies referring to motor stimulation focus on tDCS to increase motor performance,^[28–30]^ muscle endurance^[31]^ and balance.^[32,33]^ This technique has been applied to stimulate the modulation of neural tissue in motor rehabilitation and motor learning in healthy individuals, athletes and neurological and/or musculoskeletal disorders.^[34]^ tDCS allows changes in neuronal membrane potential.^[29]^ Although this brain excitability modification is not enough to generate action potentials to provide brain control on human actions,^[35]^ it induces neuron polarization which could modulate resting membrane potential.^[36]^ tDCS has been successfully used in cognitive multitasking performance.^[37]^ Indeed, the most cited articles in the literature apply transcranial stimulation to address its effects on motor activation in athletes,^[38]^ concussion in athletes,^[39]^ muscular endurance,^[23]^ motor training after stroke,^[40]^ or cognitive function after traumatic brain injury.^[41]^ Nevertheless, future studies in tDCS must refine protocols of stimulation (e.g., number of participants, inter-individual variability, duration, intensity, target and return electrode positions) to increase the impact on motor learning, motion perception, muscular strength, and fatigue, especially in expert athletes.^[4]^ In this line, tDCS should identify target brain areas of interest as it has a lower focality of an induced electric field compared to TMS.^[42]^ Angius (2018)^[25]^ reviewed 28 articles about the tDCS effects on physical performance, reporting a high variability of the results; about 60% of the studies reported positive outcomes related to physical performance, endurance, strength, power, or anaerobic work capacity. The primary motor cortex was the most targeted area, using previous stimulation to the physical task of 20 minutes at 2 mA with active electrodes of 35 cm^2^.

Scientometric analyses provide current trends in the literature within a particular area and provide rationale and incentives for future research.^[43]^ This type of analysis affords more objective and comprehensive results compared to typical literature reviews.^[44]^ Therefore, scientometrics provides quantitative, qualitative, and computational approaches to analyze the growth of one particular scientific topic.^[45]^ Bibliometric studies provide the added value of addressing relations and connections within scientific fields but also between subfields through the use of different methods for the analysis of citations.^[46]^ For example, the current scientific production on a given topic, allows the assessment of general trends in publications, researchers, journals, countries and keywords, among others.^[47]^ This information is useful when locating prominent authors, research groups or journals related to the subject and it helps to identify knowledge gaps, to support collaboration and to guide researchers to better position their work.^[48]^

Currently, there is a lack of studies about transcranial stimulation and physical activity (PA), sports performance and physical training from a scientometric perspective. In this line, this bibliometric study covers this gap, novelty showing tendencies between PA and transcranial stimulation in healthy and sports participants. To achieve this endeavor, we use some of the most popular bibliometric indicators (e.g., number of publications and citations), quantitative and qualitative methods, and science mapping techniques. The specific research questions to respond to through the bibliometric analysis would be: What are the annual publication trends on the subject?, who are the most prominent authors?, which are the most productive journals in this field?, and what are the most cited articles and the most used keywords by authors on the applications and effects of transcranial stimulation on PA and sports. Articles from the Journals indexed in web of science (WoS) will be analyzed because it is considered one of the most complete databases in the biomedical research and health sciences, using the journals as the main scientific knowledge of diffusion.^[49,50]^ We only used this database because about 99.11% and 96.61% of the journals indexed in WoS are indexed in Scopus and Dimensions, respectively.^[51]^ We anticipate that the most relevant journals and authors affiliations in the field would be placed in Western countries (Europe and America; and specifically in the Anglo-Saxon countries) because of a strong tradition in sports practices, culture about health through PA and exercise, but also a good technological support and investment in research, co-exist in these developed regions of the world to improve the quality of life and sport performance of their citizens.

## 2. Materials and methods

### 2.1. Design and data source

A mapping of research related to PA and transcranial studies was carried out using a bibliometric analysis based on bibliometrics traditional laws.^[52]^ For this purpose, all the articles and reviews published in journals indexed in the WoS Database Core Collection from Clarivate Analytic were used as a data source, restricting the search to the Science Citation Index Expanded (SCI-Expanded), the social sciences citation index, and the emerging sources citation index. The WoS is the reference database for bibliometric analysis due to the quality of the journals indexed, the prestige of its journal impact indicators (Journal Impact Factor) and its detailed information on the documents indexed.^[53–62]^ Moreover, some researchers report that using different data sources may change review outcomes because they use different criteria for assessing journal quality or author quality indicators.^[63–65]^ The following search vector was launched in WoS advanced search: (ti=(“brain polarization”) OR ab=(“brain polarization”) OR ak=(“brain polarization”) OR ti=(“neuromodulation”) OR ab=(“neuromodulation”) OR ak=(“neuromodulation”) OR ti=(“noninvasive brain stimulation”) OR ab=(“noninvasive brain stimulation”) OR ak=(“noninvasive brain stimulation”) OR ti=(“tES”) OR ab=(“tES”) OR ak=(“tES”) OR ti=(“tDCS”) OR ab=(“tDCS”) OR ak=(“tDCS”)OR ti=(“transcranial current stimulation”) OR ab=(“transcranial current stimulation”) OR ak=(“transcranial current stimulation”) OR ti=(“tDCS”) OR ab=(“tDCS”) OR ak=(“tDCS”) OR ti=(“TMS”) OR ab=(“TMS”) OR ak=(“TMS”) OR ti=(“TMS”) OR ab=(“TMS”) OR ak=(“TMS”) OR ti=(“NIBS”) OR ab=(“NIBS”) OR ak=(“NIBS”)) AND (ti=(“sport*”) OR ti=(“PA”) OR ti=(“physical training”) OR ab=(“sport*”) OR ab=(“PA”) OR ab=(“physical training”) OR ak=(“sport*”) OR ak=(“PA”) OR ak=(“physical training”)) AND (ti=(“transcranial”) OR ab=(“transcranial”) OR ak=(“transcranial”)). The search was conducted on March 8, 2023, limiting the search to articles and reviews, without other limitations (neither language, date nor access type). This search vector was intended to retrieve documents related to transcranial stimulation together with PA, sports or physical training by performing searches for documents containing these terms in their title (ti), abstract (ab) or author keywords (ak). In all the documents, the term transcranial should appear in the topic, so the ts (topic) tag was used. Table S1, http://links.lww.com/MD/K547 shows the search strategy. The dataset was downloaded from WoS in.xslx format for further processing with Microsoft Excel (Microsoft 365 MSO version 2206), and in plain text to be processed with the bibliometric analysis software VoSViewer (1.6.18). This work does not require ethics committee approval as it does not involve subjects.

### 2.2. Statistical analysis

The WoS Analyze Reports was used to check the publications temporal distribution. Subsequently, the annual publications trend was analyzed and plotted, calculating the adjustment to an exponential growth ratio (R^2^) using the exponential growth of science law from the first year in which no interruptions in annual publications were found until the present day.^[66,67]^ Lotka law was applied to highlight the prolific coauthors.^[68]^ For Lotka law application, coauthors were ordered by the number of publications, and those equal to or in a higher position than the number obtained by calculating the square root of the total number of authors were considered the prolific coauthors. A descriptive analysis to check the coauthoring countries to obtain the number of papers per country and the co-collaborative relations between them was conducted. The most productive journals were highlighted using Bradford law of concentration of science, distributing the journals into 3 Bradford zones: Core, Zone I and Zone II.^[69–71]^ The h-index was applied to identify the most cited documents, considering these as the h documents with h or more citations.^[72]^ Once the most cited papers were identified, prolific coauthors who presented at least one paper among the most cited papers were considered prominent coauthors.^[73]^ A descriptive analysis of the distribution of the documents in the WoS subject categories was carried out using WoS Analyze Reports. Finally, Zipf law was applied to highlight the most used keywords by the coauthors.^[74,75]^ The VOSviewer software was used to process the document set, running fractionalization and strength of association analyses to obtain citation or co-occurrence plots.

## 3. Results

### 3.1. Annual publications trend

**Figure 1.**
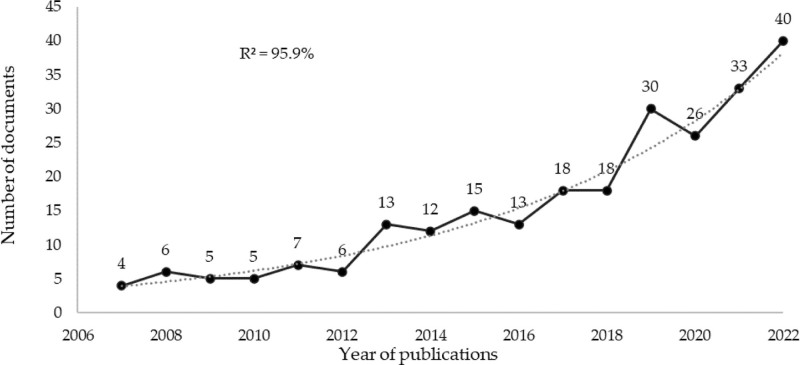
Annual publications trend.

A total of 257 documents were found (206 articles and 51 reviews). The first document was published in 1994. From 2007 to the present, there was continuity in annual publications. The trend of annual publications between 2007 and 2022 was adjusted by 95.9% (R^2^) to an exponential growth rate (Fig. 1).

### 3.2. Authors

**Figure 2.**
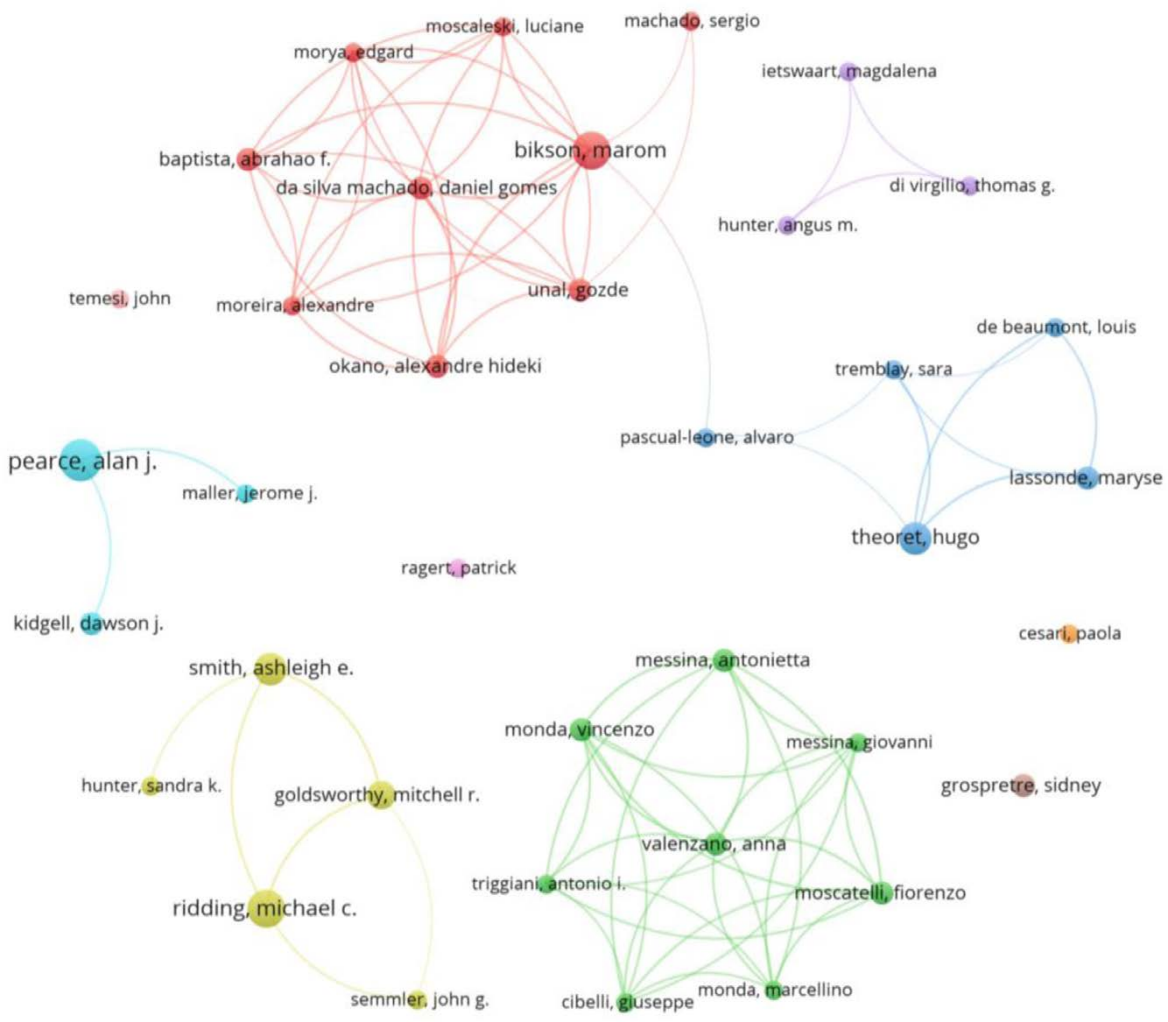
Graph with 37 prominent coauthors (analysis: fractionalization; attraction: 7; repulsion: −1; node size: documents. color: cluster).

By applying Lotka Law to the 1131 coauthors found, it was estimated that the prolific coauthors would be the 34 with the highest number of publications (square root of 1131). Thus, 17 coauthors were found with 5 or more papers, and 37 with 4 or more papers, the latter considered the prolific coauthors in the subject area. Alan J. Pearce (nine documents), Maron Bikson and Michael C. Ridding (eight documents), and Alastair D. Smith and Hugo Théoret (seven documents) were the 5 most productive coauthors on the topic. Using bio from their research websites, Alan J. Pearce specifically investigates concussion and repetitive brain trauma using noninvasive brain stimulation techniques including single/paired-pulse TMS, rTMS, and tDCS. Maron Bikson studies the effects of electricity on the human body and applies this knowledge toward the development of medical devices and electrical safety guidelines, being biomedical engineering and brain function and disease some of his areas of expertise. Michael C. Ridding does research in brain function (primarily plasticity), both in healthy and impaired populations, using cutting edge noninvasive techniques. Alastair D. Smith works on individual case and group studies of neurological patients with acquired disorders of spatial representation in various functional domains (i.e., integrative agnosia, constructional apraxia, unilateral visual neglect), and how the brain utilizes spatial information to interact with our surroundings. Hugo Théoret focuses on neuroscience, TMS, motor cortex (physical therapy), and cognition (cognitive psychology and neuroimaging). Figure 2, shows the 37 prolific coauthors and how they collaborated in co-authorship.

Cross-referencing the prolific coauthors with the most cited papers, 23 prominent coauthors were identified, prolific coauthors who contributed between 1 and 5 of the most cited papers. Among the 23 prominent coauthors, Michael C. Ridding, Hugo Théoret and Maryse Lassonde (with 5 most cited papers) and Louis de Beaumont and Sara Tremblay (with 4 most cited papers) were particularly prominent. Specifically, Maryse Lassonde drives research into brain reorganization following congenital anomalies or neurosurgery based on therapeutic purposes for children with epilepsy. She has also done clinical evaluation of the aftereffects of concussion in National Hockey League players, leading initiatives to increase the presence of women in science and engineering. The research interests of Louis de Beaumont include TMS, genetic factors influencing the recovery of patients with mild traumatic brain injury, and the effect of age on post-trauma recovery. Sara Tremblay is an expert researcher in the use of neuromodulation techniques, including TMS. Her research has been focused on the combined use of neuromodulation and neuroimaging as biomarkers of neurological conditions (e.g., sports concussions) or towards the treatment of depression. Prominent coauthors are presented in Table S2, Supplemental Content, http://links.lww.com/MD/K548.

### 3.3. Countries/regions

**Figure 3.**
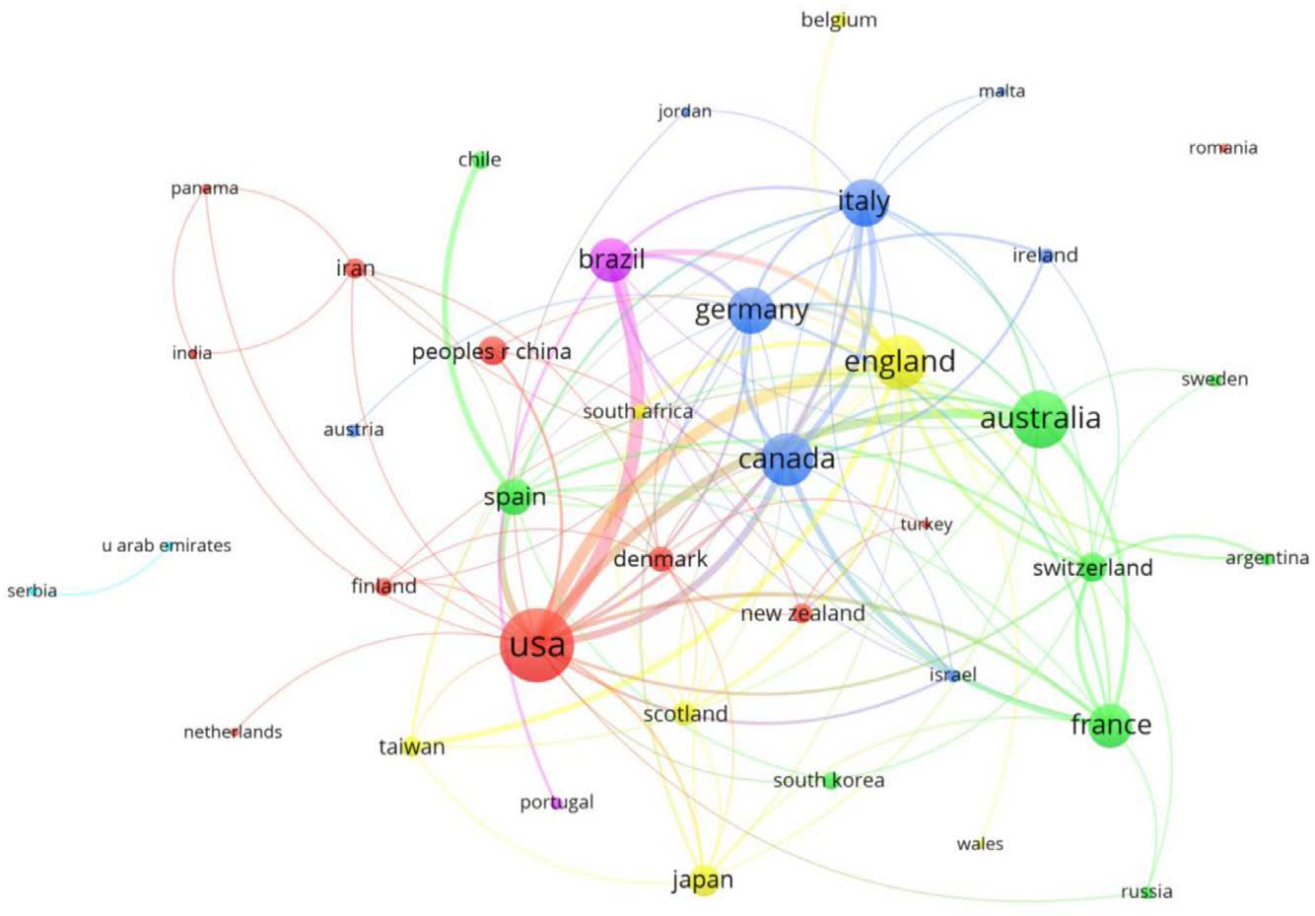
Graph with countries/regions.

The USA (67 documents) was the country/region with the highest number of documents. The other most productive countries were Australia (40), England (36), Canada (33), Italy (27), Germany (26), France (24), Brazil (23), Spain (16) and Japan (12). According to the number of citations, Italy (2083), the USA (1877), England (1349), Australia (40), Canada (1142), France (675), Germany (568), Brazil (342), Switzerland (311) and Taiwan (302) were the most cited. After performing the association strength analysis, the countries/regions appeared grouped in 4 large collaboration groups. The largest collaborative group was formed around the USA, together with Denmark, Finland, India, Iran, Netherlands, Panama, the Peoples Republic of China and Turkey. Among the countries with the most interactions with other regions, the most important were the USA (21 links), followed by England (18), Germany (15), Australia and Italy (14) (Fig. 3, Supplemental Content).

### 3.4. Most productive journals

The full set of documents was published in 133 journals. Applying Bradford law to the journals according to the number of published manuscripts, Bradford Core consisted of 14 journals accumulating 87 papers (34% of publications), 30 journals in Zone I (81 papers, 32% of publications) and 89 journals in Zone II (89 papers, 35% of publications). The distribution of the journals was adjusted to the theoretical Bradford series with an error of -6.1% (Table S3, http://links.lww.com/MD/K549). Table 1, shows the 14 journals that formed the Bradfords Core of prolific publications.

**Table 1:** Bradford core journals by the number of published manuscripts.

Bradford zone	Journals (Publisher)	Articles	%Acc	Cites	JIF	JCR	%OA
Core	Frontiers in human neuroscience (Frontiers Media)	12	5%	193	3.473	Q2	97.7%
	Medicine and Science in Sports and Exercise (Lippincott Williams & Wilkins)	11	4%	491	5.411	Q1	7.0%
	Clinical Neurophysiology (Elsevier Ireland)	7	3%	326	4.861	Q2	17.4%
	Experimental Brain Research (Springer)	6	2%	113	2.064	Q4	20.9%
	Journal of Applied Physiology (Amer Physiological Soc)	6	2%	214	3.881	Q2	5.8%
	Brain Sciences (MDPI)	5	2%	38	3.333	Q3	95.7%
	Brain Stimulation (Elsevier Science Inc.)	5	2%	199	9.184	Q1	75.5%
	European Journal of Neuroscience (Wiley)	5	2%	328	3.698	Q3	26.0%
	European Journal of Sport Science (Taylor & Francis)	5	2%	125	3.980	Q2	9.1%
	International Journal of Environmental Research and Public Health (MDPI)	5	2%	5	4.614	Q1	96.1%
	Journal of Science and Medicine in Sport (Elsevier)	5	2%	154	4.597	Q1	8.4%
	Neuroscience (Pergamon-Elsevier)	5	2%	115	3.708	Q3	10.3%
	Scientific Reports (Nature Portfolio)	5	2%	18	4.997	Q2	99.6%
	Trials (BMC)	5	2%	21	2.728	Q4	99.8%

% Acc. = percentage of accumulated documents, JCR = journal citation reports quartile, JIF = journal impact factor, %OA = percentage of open access.

### 3.5. Most cited papers

**Figure 4.**
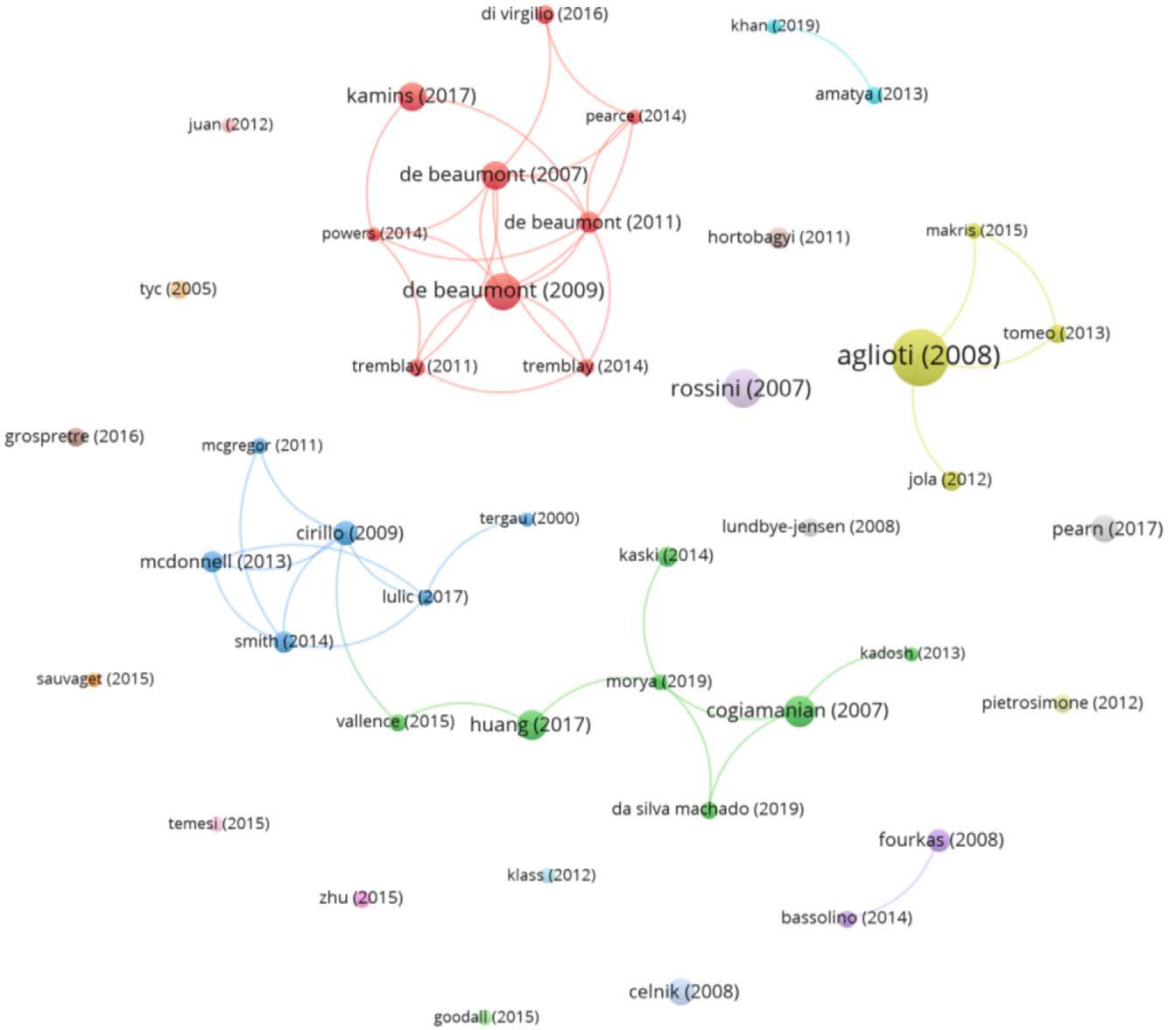
Citation network in most cited documents.

Using the h-index to identify the most cited papers, 44 papers with 45 or more citations were found. Figure 4 displays the citation analysis graph generated with the 44 most cited documents. The node size depends on the number of citations. The largest node corresponds to the most cited paper “Action anticipation and motor resonance in elite basketball players” (683 citations).^[38]^ This study published by Aglioti (2008) was cited by 3 of the most cited papers (Jola, 2012,^[76]^ Tomeo, 2013,^[77]^ and Makris, 2015^[78]^). The second most cited paper was “Clinical neurophysiology of aging brain: From normal aging to neurodegeneration” (322 citations), published by Rossini et al in 2007,^[79]^ which was not cited by any of the most cited papers. The third most cited paper, “Brain function decline in healthy retired athletes who sustained their last sports concussion in early adulthood”^[80]^ (299 citations) was cited in 5 of the most cited papers. Around this paper, together with the paper “Long-term and cumulative effects of sports concussion on motor cortex inhibition”^[39]^ (179 citations), both by De Beaumont and col., one of the largest clusters of most cited papers were found, grouping 9 papers. Among the most cited, the 3 most recent papers are “Beyond the target area: an integrative view of tDCS-induced motor cortex modulation in patients and athletes” (58 citations) published in 2019 by Morya^[34]^; “Effect of tDCS on exercise performance: A systematic review and meta-analysis” (62 citations) by Machado^[81]^; and “Non-pharmacological interventions for spasticity in adults: An overview of systematic reviews” (46 citations) by Khan.^[82]^

**Figure 5.**
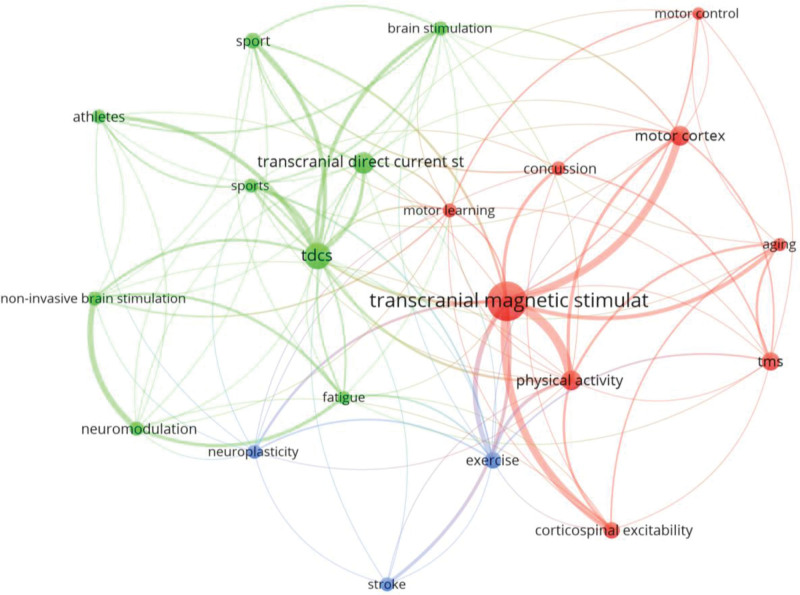
Cited reference network.

The citation analysis of all documents revealed 5 major clusters of articles (Fig. 5). The cluster with the most cited references had as reference paper: “Improved isometric force endurance after tDCS over the human motor cortical areas” by Cogiamanian et al and “Plasticity induced by noninvasive transcranial brain stimulation: A position paper” by Huang et al forming a cluster with 91 papers (blue cluster). Around the article “Clinical neurophysiology of aging brain: From normal aging to neurodegeneration,” published by Rossini et al, there was another cluster of papers with 86 cited references, including “Motor cortex plasticity induced by paired associative stimulation is enhanced in physically active individuals,” published by Cirilo et al, and “The influence of a single bout of aerobic exercise on short interval intracortical excitability,” by Smith et al (green cluster). The third largest trend, with 52 cited papers (yellow cluster), was formed around the publications by De Beaumont et al: “Brain function decline in healthy retired athletes who sustained their last sports concussion in early adulthood” and “Long-term and cumulative effects of sports concussion on motor cortex inhibition.” A fourth cluster with 29 cited papers was formed with some of the most cited papers: “What is the physiological time to recovery after concussion? A systematic review” by Kamins et al, “Pathophysiology Associated with Traumatic Brain Injury: Current Treatments and Potential Novel Therapeutics” by Pearn et al, “Improved Cognitive Function After Transcranial, Light-Emitting Diode Treatments in Chronic, Traumatic Brain Injury: Two Case Reports” by Naeser et al, among others (red cluster). Finally, the fifth trend (27 cited papers) was formed around the most cited paper of the set, together with others such as: “Effects of action observation on physical training after stroke” by Celnik et al or “Kinesthetic imagery and tool-specific modulation of corticospinal representations in expert tennis players” by Fourkas et al among others (purple cluster).

### 3.6. WoS categories

The articles were catalogued in 43 different WoS thematic categories. More than 80% were related to 3 categories: Neurosciences (42.3%, 109 documents), Sport Sciences (26.8%, 69 documents) and Clinical Neurology (12.1%, 31 documents). Other subject categories that were raised due to the number of related articles were Psychology (30 documents), Physiology (23 documents), Rehabilitation (21 documents), Multidisciplinary Sciences (11 documents), Psychology Experimental (12 documents), Medicine General Internal (10 documents) and Behavioral Sciences (9 documents), among others. The most cited papers, the prolific coauthors and the most productive journals and publishers in the 3 prominent subject categories were: “Action anticipation and motor resonance in elite basketball players,” published in Nature Neuroscience with 683 citations, M.C. Ridding (6 documents), Frontiers in Human Neuroscience (12 documents) and Elsevier (32 documents) in the Neuroscience category; “What is the physiological time to recovery after concussion? A systematic review,” published in the British Journal of Sports Medicine with 181 citations, A.J. Pearce (4 documents), Medicine and Science in Sports and Exercise (11 documents) and Lippincott Williams & Wilkins (16 documents) in Sport Sciences; and “Brain function decline in healthy retired athletes who sustained their last sports concussion in early adulthood” published in Brain, with 299 citations, M. Lassonde and H. Theoret (4 documents), Clinical Neurophysiology (7 documents) and Elsevier (14 documents) in Clinical Neurology.

### 3.7. Author keywords

**Figure 6.**
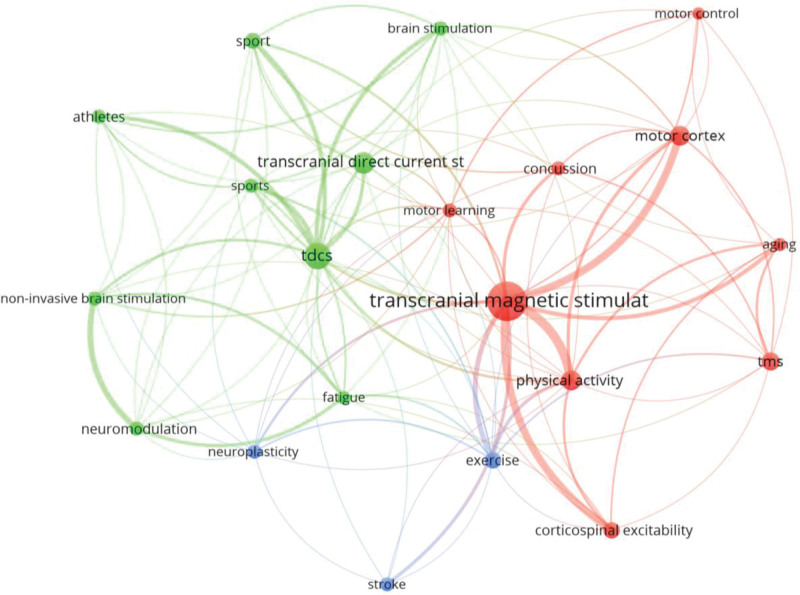
Most used keywords.

A total of 612 keywords were used by the authors. After applying Zipff Law it was estimated that prominent keywords should be 25 with the most occurrences (square root of 612). Then, 28 keywords were found with 7 or more occurrences and 21 with 8 or more occurrences, the latter being of most interest to the coauthors. The most frequently used keyword concept was “TMS” (80 occurrences) plus 18 more occurrences with its acronym “TMS.” “tDCS” (24 occurrences) or its acronym “tDCS” (36 occurrences) were also among the most used keywords together with “PA” (21 occurrences), “motor cortex” (20), “exercise” (15), “corticospinal excitability” (14), “sport” (13), neuromodulation” (11), “athletes” (11) or “concussion” (11). The most used keywords in the fractionalization analysis were grouped into the 2 largest thematic categories around the concepts: TMS and tDCS, and a third with the terms exercise, stroke and neuroplasticity (Fig. 6). Among the prominent concepts, “tDCS,” “motor learning,” “neuromodulation,” “noninvasive brain stimulation,” “neuroplasticity,” “sport” and “PA,” were the ones with the most recent publication year averages (Fig. S1, http://links.lww.com/MD/K546).

## 4. Discussion

This study presents a bibliometric analysis of transcranial stimulation and PA; to the best of our knowledge, this is the first bibliometric study covering this topic. Bradford law and different bibliometric parameters were applied to study the associations between coauthors, publications by countries/regions, keywords, and categories. 257 articles included in the WoS database were analyzed from multiple journals on transcranial stimulation and PA published between 1994 and 2023. These scientometrics data revealed it is an emerging research field with an increased number of publications in the last years. An exponential growth of the scientific production was found due to the need to search for new training techniques for the modulation of brain functionating that potentially would enhance motor and cognitive athletic performance. Empirical evidence supports the assumption that transcranial stimulation and PA is a topic of interest for the scientific community with a growing tendency of publications between 2007 and 2023 (98.4% of the total publications). Moreover, the number of papers published since 2013 was higher than the ten annual documents. As a result, when applying DeSolla Price exponential growth law of science, it was found that annual publications had an exponential growth ratio. The first publications on this topic dealt with TMS in sports injuries and changes caused by PA in motor-evoked potentials.^[83,84]^ As a result of these publications, new research sub-topics emerged (e.g., the effects of TMS on the cognitive and motor performance of non-athletes, elite players, and special populations).

There were 37 prolific authors following Lotka Law. The author with the highest number of documents was Alan J. Pearce (9 articles), followed by M. Bikson and M. Ridding (8 articles), and A. Smith and H. Theoret (7 articles). Alan J. Pearce was the most productive author with publications since 2009. His most cited article deals with the TMS effects on cognitive and fine motor activity in retired elite Australian soccer players who suffered concussions during their sports careers. Additionally, the most cited authors were S. Aglioti with 875 citations and 3 publications, C. Urgesi with 818 citations and 3 articles, and P. Cesari with 768 and 4 publications. The most cited coauthor, S. Aglioti, accumulated most of his citations in 1 paper, the most cited paper “Action anticipation and motor resonance in elite basketball players.”^[38]^ This article was published in 2008 and aimed to investigate the neural relationships in anticipation and decision-making of professional basketball players combining psychophysics and TMS. This study has a large number of citations due to the participation of elite players and implications for the preparation of athletes related to skill anticipation in the sports domain. However, they are not prominent as they have 3 papers, while M.C. Ridding, H. Theoret and M. Lassonde had 5 papers among the most cited.

Anglo-Saxon countries led in scientific production (the USA, Canada, Australia, and England) over such countries as Italy, Germany, France, and Spain. Italy achieves the highest number of citations with a low number of publications (27), and this is because the most cited authors are from this country (S. Aglioti, C. Urgesi, and P. Cesari). However, various countries have increased their attention to this research topic in the last few years, achieving a great number of citations for their publications. The cases of Japan, Peoples R. China, and Brazil are notable and have contributed to the productivity on this topic. Regarding interactions with researchers from other countries, the Anglo-Saxon and European countries seem to similarly collaborate with colleagues from other nationalities. The USA is the most active country as presents the best results in published documents and citations. Therefore, the USA leads scientific research in most scientific areas.^[85]^

After applying Bradford law, it was found that the Core was composed of 14 journals that accounted for 34% of the publications, most of them mixed (subscription or open access). The first journal in the Core was Frontiers in Human Neuroscience, Q2 in the JCR, with a 97.7% Open Access rate. This journal contributed the most papers in the WoS category “Neuroscience.” Medicine and Science and Exercise was the second most productive journal contributing with 11 documents, being Q1 in the Sport Sciences thematic category. From the 43 categories that appeared in the search, most of the articles were in the Neuroscience, Sports Sciences, and Clinical Neurology categories (81.3%). The most cited document was “Action anticipation and motor resonance in elite basketball players,” by S. Aglioti,^[33]^ located in the Neuroscience thematic category. In the Sports Sciences category, the journal with the greatest number of publications (the second in Bradford Core) was Medicine and Science in Sports and Exercise, and the prolific author was A.J. Pearce. The paper with the most citations was “What is the physiological time to recovery after concussion: A systematic review.”^[72]^ Finally, in the Clinical Neurology category, the most productive journal Clinical Neurophysiology ranked third in Bradford Core. This journal was found in Q2 of the JCR. The article with the most citations was “Brain function decline in healthy retired athletes who sustained their last sports concussion in early adulthood.”^[73]^ These journals have mixed access, although Frontiers in Neurophysiology was an Open Access journal, and Medicine and Science and Exercise was almost exclusively a subscription journal. Finally, 2 main clusters could be distinguished in the keywords, “TMS” and “tDCS”. The most cited article and the most influential author were related to the keyword “TMS.” Moreover, the most recent publications were focused on tDCS,^[74–76]^ while initial publications were more focused on TMS.^[69–71]^ However, another cluster formed by neuromodulation, exercise and stroke was found, separate from the previous clusters, and this is another line of research.^[86]^

This study has several practical applications. First, readers and researchers could benefit from relevant information published by other researchers on the topic during the past years because scientometric data provide an opportunity to evaluate the quality and quantity of the existing scientific research conducted about the treated topic. Furthermore, this study findings may be important for detecting new research ideas to handle the next research and also help in the establishment of beneficial collaborations and networks among researchers to manage correctly well-driven future scientific projects. In this line, the study findings will aid future researchers in predicting potential industry trends. For example, a new research gap to consider in the future would be the effect of brain stimulation on whole-body exercise and during competitions, rather than in controlled laboratory conditions.^[25]^ Second, the information shown in this study may support better decisions in public and/or private institutions when prioritizing funding for all projects related to this research topic. For example, if there is a relevant author or/and coauthors largely cited in the literature, the country/State/University where he/she/they worked could prime his/her/their research lines (i.e., evaluating with more scoring these projects based on the previous journal outcomes) to consolidate this international ranking position. Additionally, it could identify emerging research groups with investment needs, reinforcing their research lines with the aggregation of new scientific equipment and/or personal staff to conduct well-focused intervention designs for next project calls. For example, creating specific funding opportunities for these incipient research groups and academics that require gaining experience and knowledge in the field.

This study has some limitations as the nonuse of the Scopus database as its foundation in searching scientific publications. This could imply the exclusion of some publications. To this end, future research on this topic should extend this bibliometric analysis to other databases such as Scopus, EBSCO, and ProQuest, among others. The use of English-language journals is overrepresented to the detriment of other languages in WoS. Therefore, next bibliometric studies in PA and transcranial stimulation should include new methods and indicators from field-specific and national citation indexes to gain a more comprehensive analysis of the research topic. For example, the use of books, proceedings and reports as other means of scientific knowledge diffusion would strengthen the bibliometric analysis and provide a more comprehensive state of the art in the field.

## 5. Conclusions

Annual publications followed an exponential growth trend (R^2^ = 95.9%). Thirty-seven prolific coauthors with 4, papers were identified, with A.J. Pearce (9 documents), as the most productive coauthor. However, the prominent coauthors were M.C. Ridding, H. Theoret and M. Lassonde (with 5 most cited papers). The USA (67 documents) and Journal Frontiers in Human Neuroscience (12 documents) were the most productive country and journal, respectively. “Action anticipation and motor resonance in elite basketball players” was the most cited paper and “TMS” was the most used keyword. However, research trends in recent years seem to be more focused on the effects of tDCS. There are extensive research networks throughout the world, with the USA at the forefront of production. Publications on the subject seem to reveal a high interest in the scientific community, increasing exponentially over the years.

## Author contributions

**Conceptualization:** Noelia Mayordomo-Pinilla, Sabina Barrios Fernández, Santiago Gómez-Paniagua.

**Formal analysis:** Angel Denche-Zamorano.

**Funding acquisition:** Antonio Castillo-Paredes, Laura Muñoz-Bermejo.

**Methodology:** Angel Denche-Zamorano, Antonio Castillo-Paredes.

**Visualization:** Vicente Luis-del Campo, Jorge Rojo-Ramos.

**Writing – original draft:** Noelia Mayordomo-Pinilla, Sabina Barrios Fernández, Jorge Rojo-Ramos.

**Writing – review & editing:** Vicente Luis-del Campo, Santiago Gómez-Paniagua, Laura Muñoz-Bermejo.

## Supplementary Material
