# Supplementary material for: A scientometrics analysis of physical activity and transcranial stimulation research
Source: Medicine (Baltimore). 2023 Nov 24;102(47):e35834. doi: 10.1097/MD.0000000000035834 (PMC10681591; doi:10.1097/MD.0000000000035834)
Supplement: Supplementary file 1 [file medi-102-e35834-s001.docx]

| Table 1. Search strategy. | | | | |
| --- | --- | --- | --- | --- |
| Papers with at least one of these terms in title, abstract or author keywords. |  | Papers with at least one of these terms in title, abstract or author keywords. |  | Papers with this terms in title, abstract or author keywords. |
| Brain polarization | AND | Physical activity | AND | Transcranial |
| Neuromodulation |  | Sport* |  |  |
| Non-invasive brain stimulation |  | Physical training |  |  |
| NIBS |  |  |  |  |
| Transcranial electrical stimulation |  |  |  |  |
| Transcranial direct current stimulation |  |  |  |  |
| Transcranial current stimulation |  |  |  |  |
| tDCS |  |  |  |  |
| Transcranial magntic stimulation |  |  |  |  |
| TMS |  |  |  |  |

tDCS (Transcranial direct current stimulation), TMS (Transcranial magnetic stimulation).
