# Supplementary material for: A scientometrics analysis of physical activity and transcranial stimulation research
Source: Medicine (Baltimore). 2023 Nov 24;102(47):e35834. doi: 10.1097/MD.0000000000035834 (PMC10681591; doi:10.1097/MD.0000000000035834)
Supplement: Supplementary file 2 [file medi-102-e35834-s002.docx]

| Table S2. Prominent co-authors. | | | | |
| --- | --- | --- | --- | --- |
| Author's name | Doc. | Cit. | Most cited papers | WOS ID |
| Ridding, Michael C. | 8 | 559 | 5 | WOS:000415788100028; WOS:000272838200004; WOS:000318547200007; WOS:000336732400027; WOS:000360008800025 |
| Theoret, Hugo | 7 | 719 | 5 | WOS:000264889000014; WOS:000248525400024; WOS:000291123400003; WOS:000289240200001; WOS:000336507100012 |
| Lassonde, Maryse | 5 | 697 | 5 | WOS:000264889000014; WOS:000248525400024; WOS:000291123400003; WOS:000289240200001; WOS:000336507100012 |
| De Beaumont, Louis | 4 | 639 | 4 | WOS:000264889000014; WOS:000248525400024; WOS:000291123400003; WOS:000289240200001; |
| Tremblay, Sara | 4 | 143 | 4 | WOS:000264889000014; WOS:000291123400003; WOS:000289240200001; WOS:000336507100012 |
| Bikson, Marom | 8 | 192 | 2 | WOS:000465395800001; WOS:000497545800001 |
| Goldsworthy, Mitchell R. | 6 | 193 | 2 | WOS:000336732400027; WOS:000360008800025 |
| Machado, Daniel G. | 5 | 113 | 2 | WOS:000465395800001; WOS:000497545800001 |
| Okano, Alexandre H. | 5 | 113 | 2 | WOS:000465395800001; WOS:000497545800001 |
| Cesari, Paola | 4 | 761 | 2 | WOS:000258720000022; WOS:000325761100022 |
| Pearce, Alan J. | 9 | 236 | 1 | WOS:000338983500002 |
| Smith, Ashleigh E. | 7 | 164 | 1 | WOS:000336732400027 |
| Baptista, Abrahao F. | 5 | 95 | 1 | WOS:000497545800001 |
| Grospretre, Sidney | 5 | 88 | 1 | WOS:000372040100006 |
| Unal, Gozde | 5 | 71 | 1 | WOS:000465395800001 |
| Ainslie, Philip N. | 4 | 183 | 1 | WOS:000325987300063 |
| Di Virgilio, Thomas G. | 4 | 91 | 1 | WOS:000390704800022 |
| Hunter, Angus M. | 4 | 91 | 1 | WOS:000390704800022 |
| Ietswaart, Magdalena | 4 | 90 | 1 | WOS:000390704800022 |
| Temesi, John | 4 | 76 | 1 | WOS:000356493300007 |
| Moreira, Alexandre | 4 | 62 | 1 | WOS:000465395800001 |
| Morya, Edgard | 4 | 57 | 1 | WOS:000497545800001 |
| Moscaleski, Luciane | 4 | 57 | 1 | WOS:000497545800001 |
| Doc. (Number of documents); Cit. (Number of citations); Most cited papers (Documents amongs the most cited papers); WOS ID (Document's unique identifier in the Web of Science) | | | | |
