# Supplementary material for: A scientometrics analysis of physical activity and transcranial stimulation research
Source: Medicine (Baltimore). 2023 Nov 24;102(47):e35834. doi: 10.1097/MD.0000000000035834 (PMC10681591; doi:10.1097/MD.0000000000035834)
Supplement: Supplementary file 3 [file medi-102-e35834-s003.docx]

| Table S3. Bradford's zones and their number of journals, according to number of documents and cites. | | | | | | | | | | |  |  |
| --- | --- | --- | --- | --- | --- | --- | --- | --- | --- | --- | --- | --- |
| According to number of documents | | | | | | | | | | | | |
| Zone | Nº journals (%) | | Number articles (%) | | Acc. nº journals (%) | | Acc. nº articles (%) | | Bradford multipliers | | Journals (theoretical serie) | |
| CORE | 14 | (9%) | 87 | (34%) | 14 | (9%) | 87 | (34%) |  | | n0 | 14 |
| Zone 1 | 30 | (23%) | 81 | (32%) | 44 | (32%) | 168 | (65%) | 2.14 | | n1 | 36 |
| Zone 2 | 89 | (68%) | 89 | (35%) | 133 | (100%) | 257 | (100%) | 2.97 | | n2 | 91 |
| Total | 133 | 100% | 257 | 100% |  |  |  |  | Mean | 2.6 |  | 141 |
|  |  |  |  |  |  |  |  |  |  |  | % Error | -6.1% |
| Nº (Number); % (Percentage); Acc. (Accumulated). | | | | | | | | | | | | |
