# Supplementary figures and images for: A scientometrics analysis of physical activity and transcranial stimulation research
Source: Medicine (Baltimore). 2023 Nov 24;102(47):e35834. doi: 10.1097/MD.0000000000035834 (PMC10681591; doi:10.1097/MD.0000000000035834)

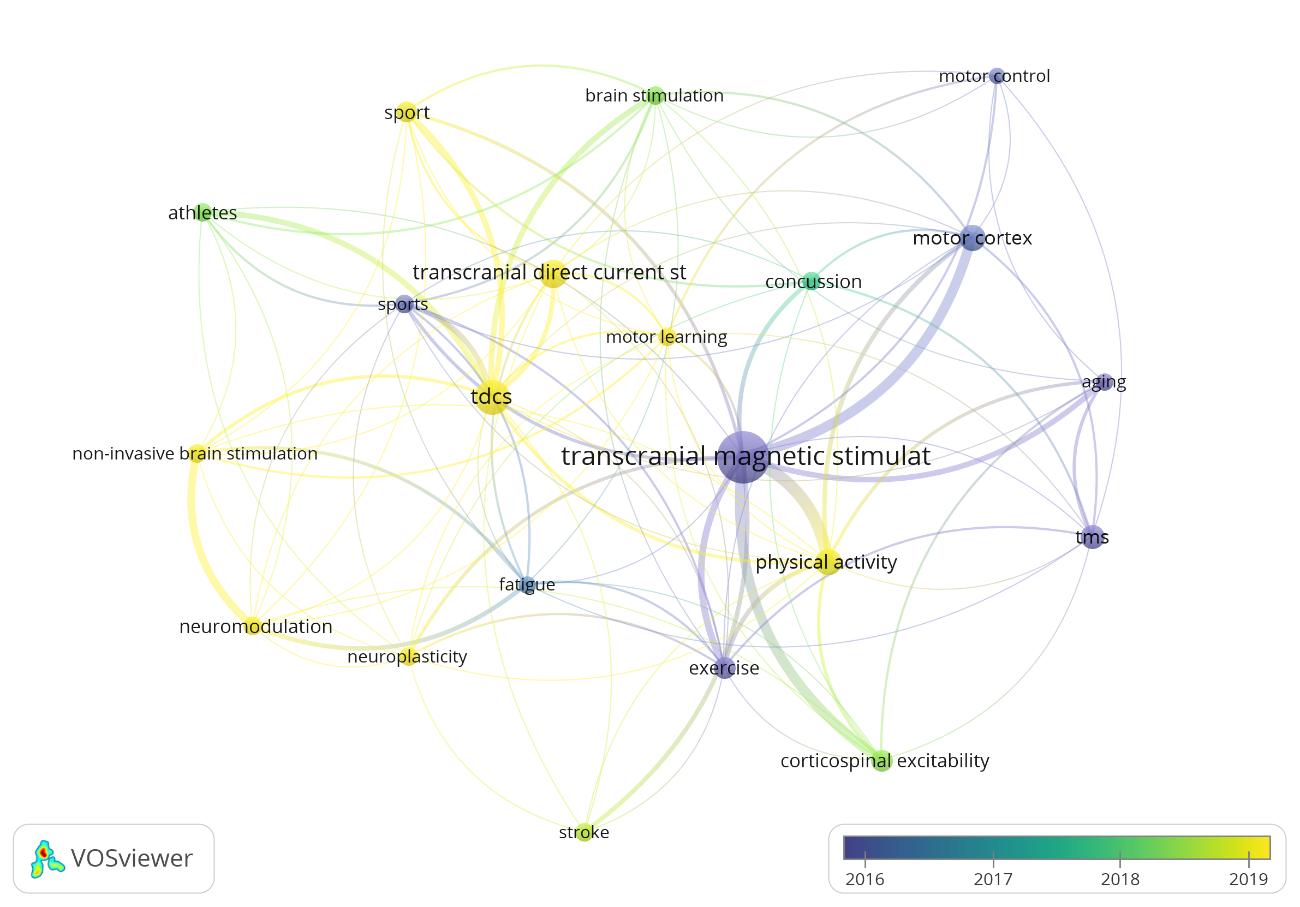


**Figure S1.** Graph with most used keywords. Score: Average publication year.

Supplement: Supplementary file 4 [file medi-102-e35834-s004.docx]
